# Vesical imaging reporting and data system (VI-RADS) in bladder cancer diagnosis in review in this number of International Brazilian Journal of Urology

**DOI:** 10.1590/S1677-5538.IBJU.2022.04.01

**Published:** 2022-07-19

**Authors:** Luciano A. Favorito

**Affiliations:** 1 Unidade de Pesquisa Urogenital Universidade do Estado do Rio de Janeiro Rio de Janeiro RJ Brasil Unidade de Pesquisa Urogenital - Universidade do Estado do Rio de Janeiro - Uerj, Rio de Janeiro, RJ, Brasil,; 2 Serviço de Urologia Hospital Federal da Lagoa Rio de Janeiro RJ Brasil Serviço de Urologia, Hospital Federal da Lagoa, Rio de Janeiro, RJ, Brasil,

The July-August number of *Int Braz J Urol* , the 17th under my supervision, presents original contributions with a lot of interesting papers in different fields: Robotic Surgery, Prostate Cancer, Overactive Bladder, Bladder Cancer, renal cancer, myelomeningocele, renal stones, congenital adrenal hyperplasia, Testicular torsion, penile cancer, BPH, Urinary incontinence and reconstructive urology. The papers came from many different countries such as Brazil, USA, Turkey, China, Belgium, Qatar and Colombia and as usual the editor´s comment highlights some of them.

In the present issue we present a important reviews about Vesical Imaging Reporting and Data System (VI-RADS) in bladder cancer diagnosis. The paper about the group of Dr.Nicola and colleagues from USA and Brazil in page 609 shows a very complete narrative review about the topic ( 1 ). The authors shows that. the technological innovation of MR imaging has advanced the assessment of bladder cancer. MR findings can be incorporated to increase the accuracy of the traditional prediction models as the EORTC, CUETO, and EAU risk stratification. The authors conclude that the use and implication of VI-RADS will improve the communication in the diagnosis, staging and surveillance of patients with bladder cancer. The editor in chief would like to highlight the following works too:

Dr. Bai and colleagues from China, presented in page 625 ( 2 ) a nice systematic review about the trifecta achievement in patients undergoing partial nephrectomy and conclude that the larger tumor size, medium and high PADUA score are associated with decreased probability of trifecta achievement.

Dr. Qin and colleagues from China performed in page 637 ( 3 ) a interesting systematic review about the Comparison of mini percutaneous nephrolithotomy and standard percutaneous nephrolithotomy for renal stones >2cm and concluded that in the treatment of >2cm renal stones, mini-PCNL should be considered an effective and reliable alternative to standard-PCNL (30FR) with less blood loss, lower transfusion rate, and shorter hospitalization. However, the mini-PCNL does not show a significant advantage over the 24F standard-PCNL. On the contrary, this procedure takes a longer operation time.

Dr. Terziotti and colleagues from Brazil performed in page 649 ( 4 ) an interesting retrospective study about the incontinence outcomes in women undergoing retropubic mid-urethral sling and conclude that both hand-made synthetic sling (HMS) and SafyreTM have similar satisfaction and subjective cure rates, with marked International Consultation on Incontinence Modular Questionnaire for Urinary Incontinence Short Form (ICIQ-UI SF) score improvement. Higher rates of intraoperative bladder injury were seen in patients who received SafyreTM retropubic sling.

Dr. Macedo Jr. and colleagues from Brazil performed in page 672 ( 5 ) a prospective study about in utero myelomeningocele repair and high-risk bladder pattern and concluded that early urological treatment of high-risk bladder pattern was effective in approximately 60% of the patients.

Dr. Diao and colleagues from USA performed in page 679 ( 6 ) a nice study about the signs and symptoms of artificial urinary sphincter cuff erosion and concluded that artificial urinary sphincther cuff erosion most commonly presents as localized scrotal inflamation symptoms. Obstructive voiding symptoms and worsening incontinence are also common. Any of these symptoms should prompt further investigation of cuff erosion.

Dr. Moschovas and colleagues from USA performed in page 696 the cover paper of this number ( 7 ). The authors eport a multicentric opinion of referral centers on different techniques to approach the Vinci single port robotic-assisted radical prostatectomy (SP-RARP) and concluded that several techniques of SP-RARP have been reported in the literature. The authors performed a multicentric collaboration describing and illustrating the most challenging steps of this surgery. We believe that the details provided in this article are useful teaching material for new centers willing to adopt the SP technology.

Dr. Elifranji and colleagues from Doha – Qatar performed in page 706 ( 8 ) the description of a interesting surgical technique to cover and fix detorsed testis undergoing fasciotomy of tunica albuginea and concluded that the orchio-septopexy after testicular fasciotomy is a simple and fast technique that can be utilized in cases of prolonged testicular ischemia and questionable viability. More than half of the testes recovered, encouraging us to propose its utilization as well as its validation by other surgeons.

The Editor-in-chief expects everyone to enjoy reading.
